# Benefits of migration in a partially-migratory tropical ungulate

**DOI:** 10.1186/1472-6785-13-36

**Published:** 2013-09-30

**Authors:** Nicolas Gaidet, Philippe Lecomte

**Affiliations:** 1 CIRAD-ES, UPR AGIRS, Montpellier, France; 2 CIRAD-BIOS, UMR CIRAD/INRA SELMET, Montpellier, France

**Keywords:** Partial migration, Habitat–performance relationships, Density dependence, Impala, Individual variability, Spatial heterogeneity, Diet quality, Savannah

## Abstract

**Background:**

Partial migration, where one portion of a population conducts seasonal migrations while the other remains on a single range, is common in wild ungulate populations. However the relative costs and benefits associated with the distinct strategies adopted by coexisting migrant and resident individuals have rarely been investigated. Here we compare the body condition of migrants and residents in a partially migratory population of impalas (*Aepyceros melampus*) in Zimbabwe. The study was conducted during two consecutive years with highly contrasted population densities (16.4 and 8.6 indiv/km^2^) due to harvesting.

**Results:**

We first identify a population substructure with a north–south sub-division in two spatial units related to distinct soils and vegetation cover. Impalas in the north range had a consistently higher diet quality and body condition than those in the south range. At the beginning of the dry season about one third of the individuals migrated from the lower (i.e. south) to the higher (i.e. north) diet quality range. This partial migration pattern was consistent between the consecutive years, and most individuals showed constancy to their moving strategy (migrant or resident). In both years, these migrants had a significantly higher body condition at the end of the dry season than the south residents that remained year-round in the lower diet quality range. Diet quality and body condition of impalas were higher in the year of lower density; however we did not detect any evidence for density-dependence in migration propensity, at the individual or population levels, nor in the benefit associated with migration.

**Conclusions:**

Our findings provide rare evidence for a significant relationship between body condition and seasonal migration strategy in wild ungulates in relation to a difference in the quality of resources acquired between distinct seasonal ranges. This study also constitutes rare evidence of partial migration in a tropical ungulate population.

## Background

In spatially and seasonally heterogeneous environments, individuals have access to resources of different quality if limited dispersal or prospection abilities, social constraints, attachment to maternal environment or ecological barriers prevent them to be distributed proportionally to resource quality and availability [1-5]. Within populations of free-ranging herbivores, the individual differences in the capacity to access and acquire forage resources contribute to the inter-individual variations in phenotypic quality and performances including lifetime reproductive success [1,5-8].

Seasonal migration is a large-scale habitat selection strategy frequently adopted by wild ungulates as a response to spatial variation in seasonal resource limitation [9-11]. In many ungulate populations, only a fraction of the population migrates between distinct seasonal ranges while the others remain sedentary year-round (i.e. partial migration [12]). This difference in strategy to track resources among co-existing migrant and resident individuals may contribute to shape individual variations in performance. Partial migration studies in ungulates have mostly focused so far on the characterisation of the biotic and abiotic differences between seasonal ranges used by residents and migrants to identify the determinants of the migration strategy (e.g. [13-15]). Incentive for migration has been most commonly related to access to higher forage quality [11,16-20] and, in some cases, to a reduced predation exposure [21,22] or human disturbance [16]. However, comparative measures of the relative performances of migrant and resident individuals have rarely been conducted to evaluate the costs and benefits associated with distinct migration tactics [17,18,23].

In this study we investigated among-individual variations in nutrition and body condition in a partially migratory population of impalas (*Aepyceros melampus*) in a semi-arid savannah in Zimbabwe, Southern Africa. Nutrition and body condition are major determinants of survival and reproductive success in ungulates [24,25] and are, therefore, commonly used proxies of individual performance. Impala is a common medium-sized African ungulate with a relatively small home-range (c. 0.5-5 km^2^[26-29]). This species is highly gregarious but has a typically unstable social group system [30]. We here tested the existence of individual variations in body condition in relation to differences in resource acquisition between impalas that used different seasonal ranges and different migration strategies. We evaluated differences in the quality of food intake using two conventional faecal indicators (i.e. the nitrogen and the fibre concentration in the faeces) related to the crude protein content and the digestibility of the diet [31,32].

**Figure 1:**
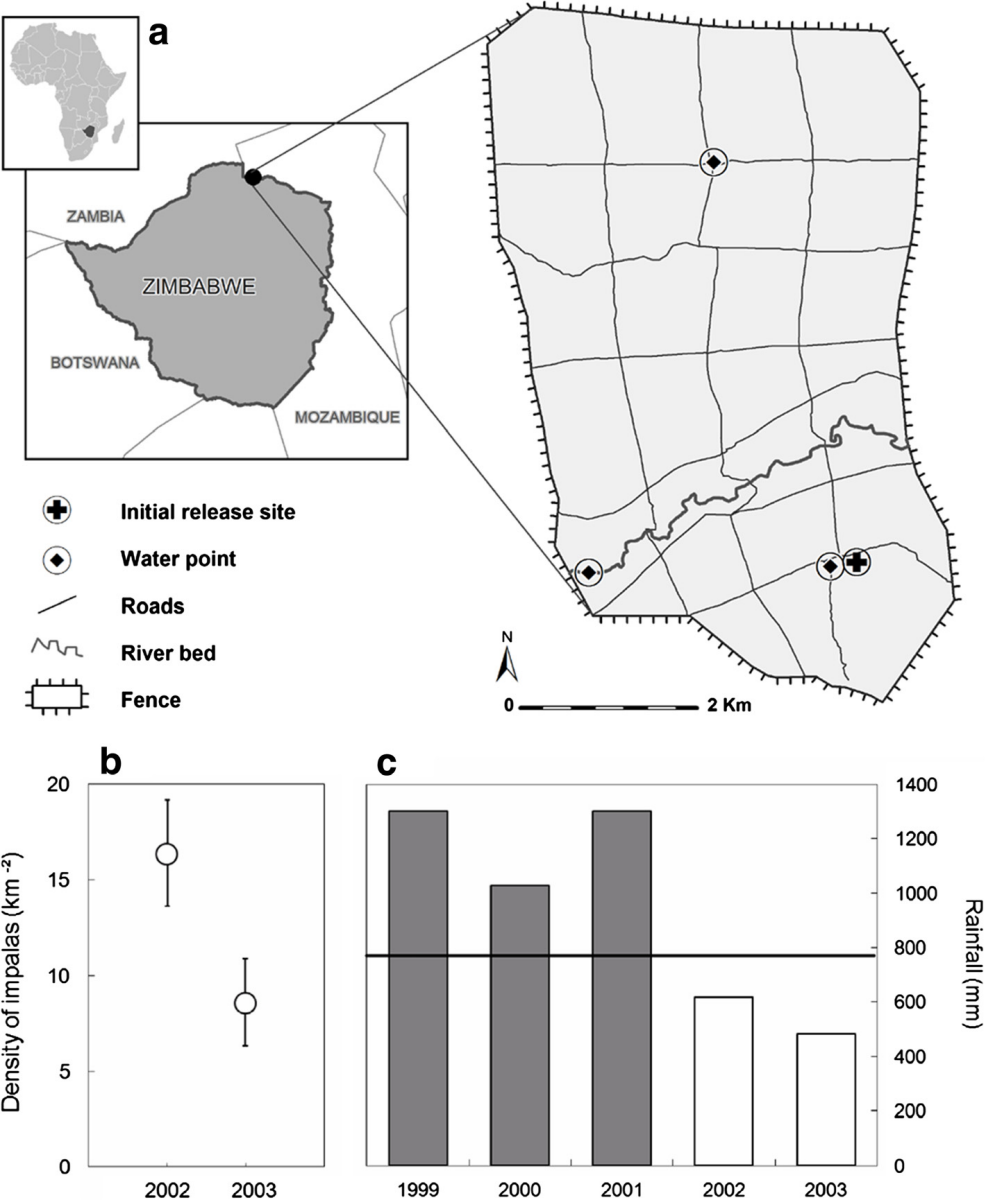
**Spatial and temporal characteristics of the study area. a)** Location and landscape features of the Chivaraidze Communal Game Ranch in northern Zimbabwe; **b)** Density of the impala population during the study period (2002-2003) estimated from the proportion of marked animals through capture-mark-recapture modelling (mean ± 95% CI; see [31]); **c)** Amount of annual precipitations from the time of introduction of impalas in the study area (1999-2000) to the study period (2002-2003); the long-term average annual rainfall (solid line: 1981-2003) is provided for reference (Zimbabwean Department of Meteorological Services, Harare).

Semi-arid tropical environments are characterised by highly seasonal rainfall, resulting in a strong reduction in the quality and the availability of forage resources during the dry season. We monitored body condition at the end of the dry season - a period of nutritional stress - when individual variations in body condition are expected to be the most contrasted. The study was conducted on a game ranch where the density of the indigenous population of impalas had been initially set high through introduction of animals to meet harvesting objectives. This impala population experienced a c.50% reduction in density between the two years of the study period, mainly due to harvesting (hunting and poaching). In a previous study we found that this drastic reduction in density resulted in a significant increase in the body condition and the reproductive performance of the population [33]. This between-year drop in density, during a period of low and relatively similar rainfall (Figure 1), provides the opportunity for studying the density-dependence response in migration propensity and associated benefits at a population scale while controlling for rainfall. According to the resource limitation hypothesis [9-11], the intraspecific competition for limited food resources should increase with density. This may result in a stronger spatial variation in resource limitation at high density. We first predict that the diet quality of impalas will be lower in the year of higher density in accordance with the lower body condition and recruitment that we previously reported [33]. We also predict a more contrasted spatial variation in diet quality and body condition in the year of higher density between impalas that used distinct seasonal ranges or migration strategies. We finally predict a higher proportion of migrants in the year of higher density if intraspecific competition promotes migration [12].

## Methods

### Study site

**Figure 2:**
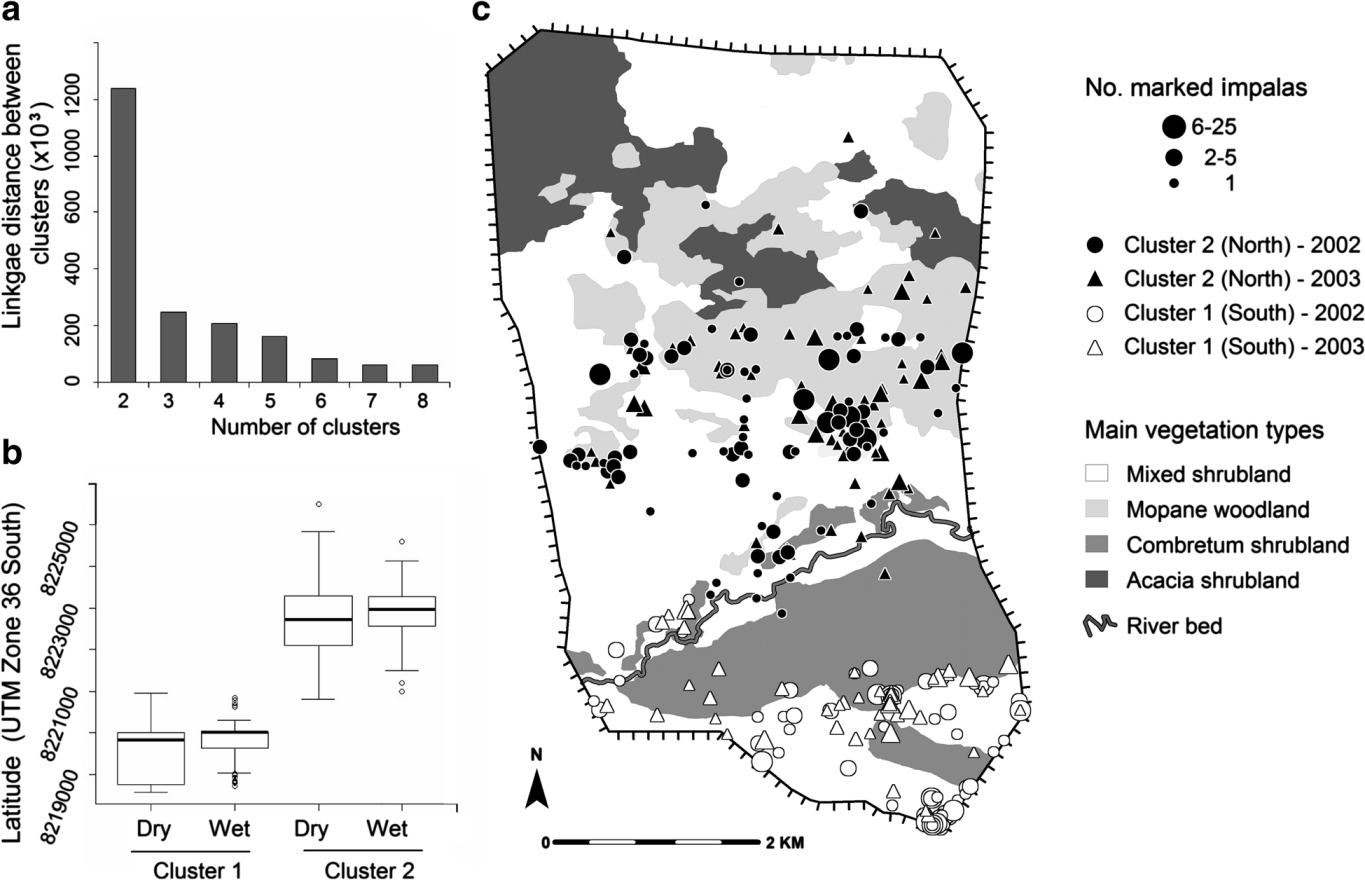
**Characterisation of the impala population sub-units.** The spatial structure has been identified by a hierarchical clustering analysis applied on all the locations of marked impalas monitored over the study area during the study period (2002 and 2003); **a)** distribution of the cluster criterion value (i.e. the dendrogram node height) in relation to the numbers of clusters; **b)** box plots of latitude values (interquartile range and outliers around the median) of locations of impalas for the two spatial clusters in each season (one unit along the y axis is equivalent to 1000 m); **c)** distribution of locations assigned to the two spatial clusters (north and south units). Group size illustrates the number of marked impalas when observed in a common herd.

The study was conducted at the Chivaraidze Communal Game Ranch located in the middle Zambezi Valley in northern Zimbabwe (Figure 1). This ranch of 3,200-ha lies on an ancient flood plain, at an altitude of 400 m. It is enclosed by a 3 meter-high electric fence erected in 1999. A systematic square grid-network (c. 1km/1km) of dust roads have been opened to provide easy access to the entire area (Figure 1). The area is covered by a mosaic of dry and deciduous woodland and shrubland consisting of four main vegetation types with contrasting physiognomy and floristic composition (previously described by [34]). A SPOT-4 satellite image and aerial photographs were used to map the distribution of these four vegetation types over the study area (Figure 2); these were validated through ground observations of vegetation cover [35].

Rainfall is highly seasonal and annually highly variable (mean: 770 mm/year, min.-max.:355-1,300 mm/year over 1981-2003, Zimbabwean Department of Meteorological Services). We considered two major seasons: a wet season from November to May corresponding to the period of major rainfall and vegetation growth, and a dry season from June to October corresponding to the period of vegetation senescence and restricted water surface availability. During the wet season drinking water was available from various water sources (pond, water points, river pools) spread over the entire ranch that filled up with local rainfall. The availability of drinking water at all these water points was monitored every month from May to October. At the end of the dry season (i.e., in October) surface water was restricted to only three and two water points in 2002 and 2003 respectively (the water point located on the river bed being dry in October 2003) (Figure 1). Predation was not measured directly, but the presence of potential predators (mainly leopard *Panthera pardus*, caracal *Felis caracal* and side-striped jackal *Canis adustus*) was assessed during the study period through spoor detection and direct observation on the network of dust roads (60 km over the entire ranch) continuously covered for management and field research activities (at least twice a week) [33].

### Study population

A small resident population of about 100 impalas was originally present in the study area as estimated from line-transect counts [36]. A total of 402 impalas were introduced in 1999 and 2000. All introduced individuals were marked with a numbered ear tag and were released after a few weeks of captivity in a boma (acclimatization enclosure) situated in the south of the ranch for adaptating to their new environment (Figure 1). The study was conducted from January 2002 to October 2003, by when all marked impalas were adults (≥ three years old). The population density of impalas estimated at the end of the dry season (October) from the proportion of marked animals observed at water points decreased from 16.4 ind./km^2^ in 2002 to 8.6 ind./km^2^ in 2003 (Figure 1) due to a combination of hunting, poaching and potential emigration [33].

### Individual seasonal ranges

The location of marked impalas was monitored through direct observation every month (5 days per month on average) from January 2002 to October 2003. The entire study area was systemically prospected by bicycle along the dense road network to locate marked impalas. Location coordinates were recorded using a GPS and mapped through a GIS. Individuals were identified by their tag number using a telescope (20-60 × 80). The utilisation a telescope with a tripod for reading individual tags requires animals to stand still. The study population in this game ranch was hunted and impalas were shy. Hunting was conducted throughout the study area from a car at night (using a spot light) along the road network. As a result, impalas routinely flee when approached by the observer and the tag ID of marked impalas observed during the prospection was often not identified. The relatively low number of locations collected for each individual per season prevented us from using conventional home range metrics to characterise individual distribution patterns. Previous studies of the social structure of impalas have identified a clear sub-division of the population into stable clans but with unstable social groups characterised by frequent fission and fusion events [28,30]. These studies revealed that the centre of activity of impalas from the same clan are strongly clustered, with an extensive overlap (c. 75% on average) between home ranges of clan members and a low overlap (c. 4%) between individuals from adjacent clans [28].

We here used a spatial cluster analysis method to characterise the discontinuous distribution of the study population and identify the subdivision units. We used a hierarchical clustering with the Ward’s agglomeration method using Euclidian distance between location coordinates of all marked impalas (‘stats’ R package, ‘hclust’ function). This sums-of-squares agglomerative clustering is based on the minimisation of within-cluster versus between-cluster variance. The distance between the nodes of the hierarchical tree was used to identify the relevant number of spatial clusters. The cluster analysis was conducted on all the locations collected during the study period (2002 and 2003) (Figure 2). Note that observations collected at water points during the body condition monitoring (see below) were not included in the spatial cluster analysis to avoid bias related to the attractive effect of water points during the dry season.

We analysed the consistency of assignment to a spatial cluster for each individual within and between seasons to evaluate the spatial fidelity to a seasonal range. A seasonal range could be attributed to only a portion of individuals from the marked population in each season and in each year since our monitoring system based on opportunistic observations did not systematically provide locations for all individuals. An impala was defined as resident when it was located in the same range in both wet and dry seasons and was defined as migrant when it used a distinct seasonal range. We categorised the direction of migration as the bearing from the wet season range to the dry season range.

We analysed the variation in the proportion of migrants between years and between males and females using a Generalized Linear Mixed Model (GLMM) with individual migration status as a binary-dependent variable (migrant vs. resident) and year and sex as fixed effects. The model was fitted with a binomial error distribution and a logit link function (lme4 R package, glmer procedure). A migration status could be attributed in the two consecutive years for about half the individuals that we monitored. To account for the effect of pseudo-replication associated with repeated measures from the same individual we included individual identity as a random effect in models. We used a likelihood ratio test (LRT) to test the significance of each variable in the model, computing the *χ*^2^ of the LRT between the model retaining and the model excluding the variable. We used the Pearson *χ*^2^-test to assess the goodness-of-fit of the selected models.

### Diet quality

We evaluated the diet nutritional quality of impalas through the analysis of the nitrogen and acid detergent fibre concentrations in the faeces. Faecal nitrogen (FN) is a widely used indicator of diet quality in ungulates reflecting (at least broadly) crude protein content of the diet [32]. Acid detergent fibre (ADF) that reflects mostly the ligno-cellulose fraction of the diet is inversely related to forage digestibility hence nutrient accessibility [31].

Faecal droppings of impalas were collected over a three week-period on three occasions each year: in the wet season (late March - early April), and at the beginning (July) and end (October) of the dry season. Samples were collected at various sites distributed over the range where marked impalas had been located. Coordinates of the sampling sites were recorded using a GPS. An average of 75 samples of fresh faeces was collected per sampling occasion. Each sample consisted of 10 to 25 pellets collected from an isolated dropping considered to represent a distinct individual. Morphological characteristics of faecal pellets permit droppings of impalas to be distinguished from those of other ungulate species present in the study area. Just after collection, faecal samples were dried at 60°C for 48 hours and mill-ground into a homogeneous powder.

Faecal samples (n = 447) were analysed separately for FN and ADF concentrations using the near infrared reflectance spectroscopy (NIRS), a cost-effective method increasingly used to measure nutritional content of free-ranging herbivores in particular ruminants [37]. NIRS estimation of FN and ADF concentrations relies on a specific calibration equation between spectral and chemical measurement from a subset of reference samples. Near infrared absorbance spectra were collected for all samples on a monochromator (NIRS 6500, Foss NIRSystems, Laurel, MD, USA) every 2 nm in the 1102 to 2498 nm range. A representative subset of 20% of the samples (n = 89) were selected among the data set based on the Mahalanobis distances between samples. The selection procedure was performed using the SELECT procedure in WINISI software (WINISI III, Infrasoft International State College, PA, USA). These reference samples were analysed for FN concentration using the Dumas elementary analysis and for ADF concentration using the Dosi Fibre method [38]. Concentrations were expressed in percentage of faeces dry mass. A multi-linear regression model was established for each constituent between the spectral absorbency data and the chemical values of the reference samples, according to a partial least squares regression procedure. The coefficients of determination of the calibration equation indicate a good predictive capacity of the model (R^2^ = 0.94 and 0.95 for FN and ADF respectively; standard errors of calibration (SEC) = 0.61 and 1.68 for FN and ADF respectively) [37]. The model performance was evaluated with cross validation procedures on 6 groups. The standard errors of cross validation (SECV) indicate a high degree of precision in the estimates of the concentration of both constituents (SECV = 0.78 and 2.37 for FN and ADF respectively), with highly significant coefficients of determination of the cross validation (mean R^2^ = 0.90 and 0.90 for FN and ADF respectively). Finally the performance to deviation ratio (RPDcv = SD/SECV) was satisfactory according to the criteria of [38] (3.2 and 3.1 for FN and ADF respectively). The predictive models built on reference samples were used to estimate FN and ADF for all faecal samples collected.

An analysis of variance (Anova) was used to test for the effects of year, season and range (i.e., the north and south population spatial units identified in the previous spatial cluster analysis) on the concentration of FN and ADF. A potential difference in the effect of range between years or between seasons was also investigated (two-way interactions between range and year and between range and season). A backward stepwise selection procedure was used with successive removals of non-significant interactions and factors. Residuals were tested for normality using the Shapiro-Wilk test.

### Body condition

The body condition of impala was estimated through visual assessment at the end of the dry season when animals were at their lowest annual condition. Body condition was estimated by the same observer (NG) and at the same period on both years (second and third week of October) for comparative purposes. Observations were conducted from a blind or a tree-platform established near water points, offering prolonged and clear observation of animals in open area and at a close range (30-50 meters). A preliminary calibration session was conducted in September 2002 to test the visual scoring criteria on the impala population. Care was taken to classify animals only when observed from the side and standing on flat ground, since sunlight incidence and muscle solicitation while bending on sand ridges for drinking could affect body appearance, hence visual scoring [39]. All the water points (three in 2002 and two in 2003) were successively monitored from 6 am to 6 pm for two to four consecutive days. Visual Body Condition (VBC) was assessed in two categories using criteria of progressive depletion of fat deposit on the animal’s hindquarters adapted from [40]. Impalas were first identified from their ear tag number and then scored as “fair” (roundness or slightly angular appearance of rump and flank) or “poor” (side view of the tail, strongly angular to extremely prominent appearance of the lumbar vertebrae or the exterior line of the ischium).

The repeatability of VBC scoring was evaluated using the Cohen’s Kappa statistic for individuals that were independently re-scored during consecutive days in each year. GLMMs were also used to analyse individual variation in body condition, with VBC score as a binary-dependent variable (poor vs. fair). About one third of impalas were scored for body condition in both years, therefore the individual identity was included as a random factor in models to account for the repeated measures. Our analysis consisted of two steps. We first tested for the effect of the dry season range (north versus south), year and sex on body condition. We tested the interaction between range and year to evaluate the potential change in the between-range differences between the two consecutive years of high and low density. In a second step we investigated whether individual variation in body condition was related to their migration strategies. We similarly tested for the effect of the migration strategy, year, sex and the interaction between migration strategy and year on body condition. For all models a likelihood ratio test (LRT) was used to test the significance of each variable in the model. We used the Pearson *χ*^2^-test to assess the goodness-of-fit of the selected models.

## Results

### Seasonal range and migration patterns

The prospection of the study area provided a total of 480 locations from 171 individual impalas (144 females and 27 males) during the first year and 618 locations from 165 individuals (128 females and 37 males) during the second year. The cluster analysis of the spatial distribution of all these locations revealed a clear partition into two spatial units, with a drastic drop in the cluster criterion value found after the two-cluster level (Figure 2a). The distribution pattern of the two spatial units showed a north–south subdivision in both wet and dry seasons (Figure 2b). The range of the north unit was dominated by a mopane woodland and the range of the south unit was dominated by a mixed woodland (Figure 2c). The presence of predators (leopard, caracal and side-striped jackal) was detected by spoor or directs observation on both spatial units. These two spatial units were located on both sides of the river bed that cross the study site, separated by an area of mixed shrubland where impalas (both marked and unmarked individuals) were rarely observed. The same north–south subdivision was observed in both years and was therefore retained to investigate individual distribution patterns. In both years, a permanent water point was available in both the north and the south spatial units (Figure 1).

Each individual was located between one to ten times per season. Individuals were seen on average on a similar number of occasions in the north range (mean = 1.94 locations per individual per season) and in the south range (mean = 2.19 locations). All the locations from an individual recorded within a given season were generally located in the same spatial unit. The consistency of individual assignment to the north or the south range within a given season was confirmed for almost all impalas (96%, n = 55 individuals) for which five to ten locations could be collected during a single season. Only two females were observed concurrently in the two distinct ranges within the same season. This high consistency in the location of individuals at the range scale justifies the attribution of a seasonal range for individuals that could be observed on only one occasion per season.

We also found a good agreement between the individual assignment to a spatial unit and the water point where these individuals were observed during the body condition monitoring. In both years all observations of marked impalas recorded at the water point located in the north of the study site (n = 93 individuals) corresponded to individuals that had been independently classified in the north-range unit for the dry season. Likewise all observations recorded at the water point located in the south (n = 56 individuals) correspond to individuals that had been classified in the south-range unit. In 2002 most observations (n = 228 individuals) were collected at the water point located on the river bed (Figure 1) where impalas from both the north and the south ranges were observed.

**Figure 3:**
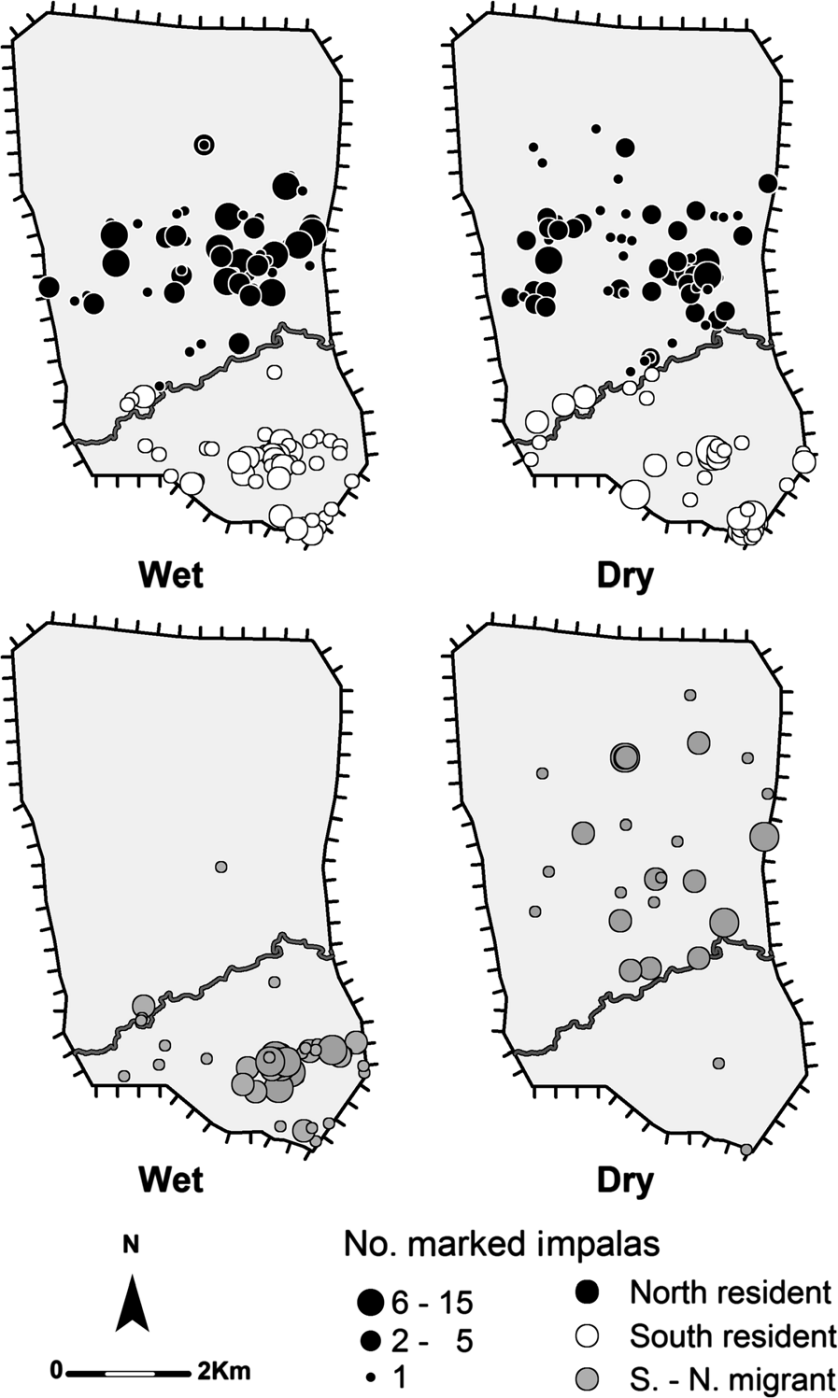
**Locations of resident and migrant impalas during the wet (November-May) and the dry (June-October) seasons.** These maps illustrate the consistency or shift in seasonal range of individuals according to their migration strategy.

**Figure 4:**
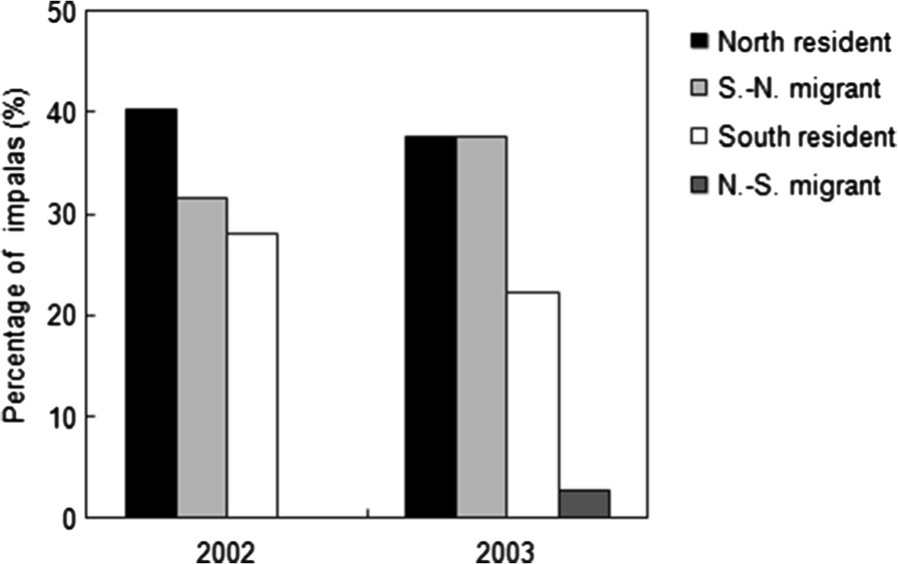
**Annual proportions of resident and migrant impalas.** No difference was found in the proportion of migrants between the years of higher (2002, n = 105) and lower (2003, n = 85) population density.

The between-year fidelity to a seasonal range was high among individuals that could be monitored in the same season both years: a large majority of individuals used the same wet season range (88%, n = 91) or the same dry season range (88%, n = 65) during the two consecutive years. Conversely, a change in the range used between the wet season and the dry season was relatively common (Figure 3). About one-third of impalas monitored in the first year (n = 105) and in the second year (n = 85) were considered migrants (Figure 4), almost all of them moving from the south to the north range between the wet and the dry seasons (S.-N. migrants, Figure 3). The first observations of migrants in their dry season range were collected in June or July. During the following wet season, almost all migrants were observed back in their previous wet season range in the south. The first observations of migrants in their wet season range were collected after the first rains in November. All other individuals were observed year-round in the same range and were considered north or south residents (Figure 3). Impalas were observed to form mixed groups of migrants and residents in both the north and the south ranges.

Our analysis of the variation in the proportion of migrants using a GLMM method revealed that most of the variance in migration status was related to differences between individuals, with a strong effect of repeated measures for individuals monitored in both years (variance estimation of the individual id. random effect = 74). Most of these individuals show a constant migration strategy over the two years: 45 out of 51 of impalas classified as resident in the first year remained resident in the second year, and 9 out of 10 impalas remained migrants (constant south to north migration). Because of lack of convergence of the GLMM (potentially due to the large number of levels within the random factor and the low number of repetitions, maximum 2 years, within each level), we re-ran the analysis using a fixed generalized linear model (GLM) on a partial data set where individuals that had been monitored in both years were randomly selected from one year only. Contrary to our prediction, we found no significant difference in the proportion of migrants between 2002 (31.6%) and 2003 (40.3%) (LRT: *χ*^2^ = 1.63, p = 0.20; difference of 0.54 ± 0.42 on a logit scale) (Figure 4). Further, no difference in the proportion of migrants was found between females (35.8%) and males (32.7%) (LRT: *χ*^2^ = 0.12, p = 0.73; difference of 0.21 ± 0.64 on a logit scale) (null model: AIC = 135.31; Pearson *χ*^2^ goodness-of-fit test = 103.0, ddl = 102, p = 0.45).

### Diet quality

**Figure 5:**
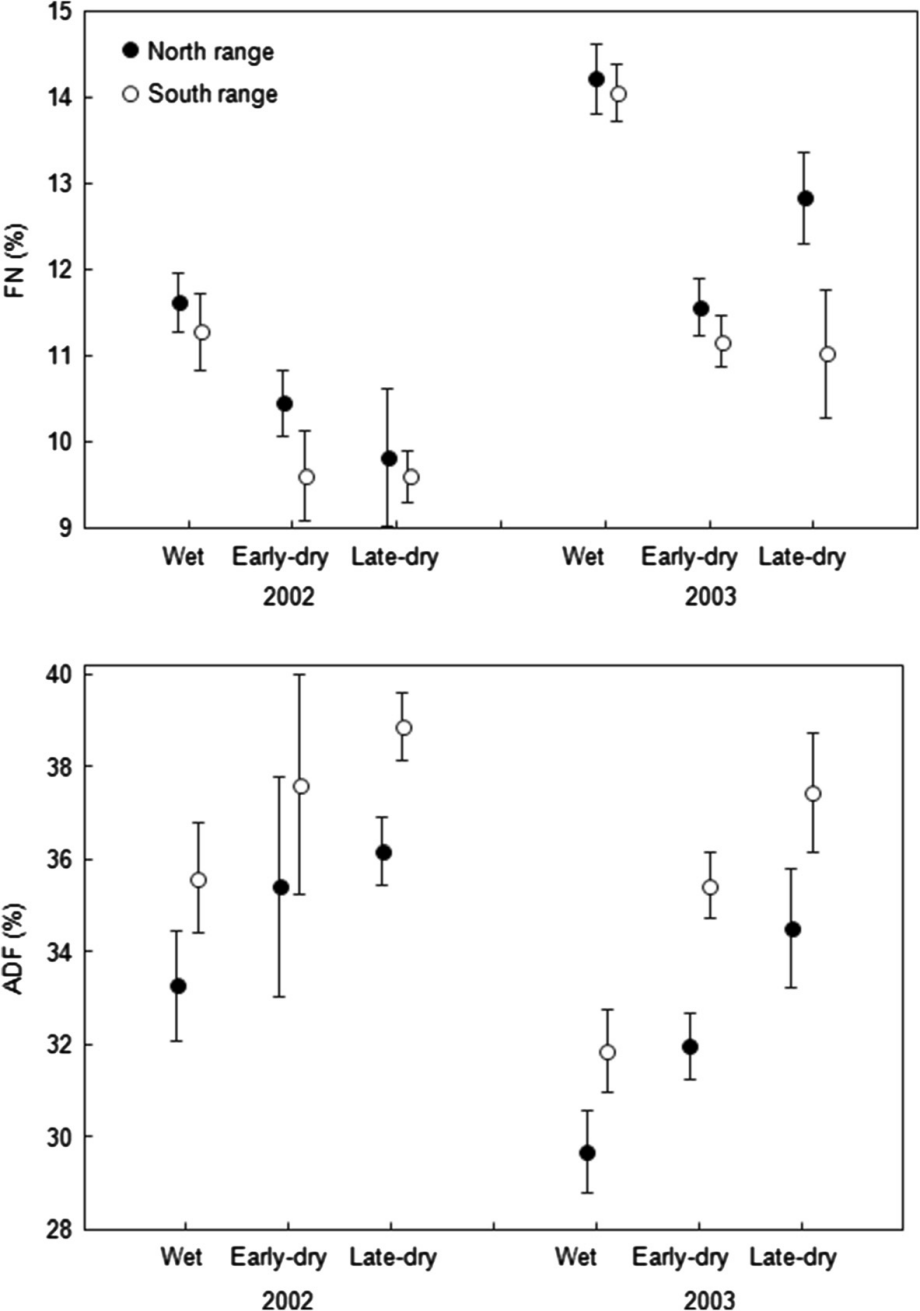
**Spatio-temporal variations in faecal nitrogen (FN) and acid detergent fibre (ADF) contents.** FN and ADF concentrations (mean ± 95% CI, as % of dry weight) were compared between the north and south ranges used by marked impalas in relation to seasons in years of higher (2002) and lower (2003) population density. A three-way anova revealed significant main effects of year, season and range (P < 0.001; see text for details).

We found a seasonal reduction in diet quality (Figure 5) with a significant decrease in nitrogen concentration (FN) and a significant increase in fibre concentration (ADF) between the wet and the dry seasons (Anova, Season: F = 118.22, p < 0.001 for FN; F = 43.36, p < 0.001 for ADF). We also found a significant difference in diet quality between years, with a higher nitrogen and a lower fibre concentration in the second year when impala density was lower (Year: F = 191.15, p < 0.001 for FN; F = 49.00, p < 0.001 for ADF).

We classified the faeces sampling sites from their coordinates into the north and south ranges. We found a significant difference in nitrogen and fibre concentrations between the two ranges (Range: F = 4.45, p < 0.001 for FN; F = 32.32, p < 0.001 for ADF). The diet quality was higher in the north than in the south range in all seasons and in both years (interactions between range and year or between range and season were not significant) (Figure 5). All these relationships remained significant after removing outliers (mean ± 2 standard deviations) to satisfy the assumption of normality of residuals (Shapiro–Wilk, W = 0.99, p = 0.16 for FN; W = 0.99, p = 0.48 for ADF).

### Variation in body condition

Body condition was assessed for a total of 153 individuals (126 females and 27 males) during the first dry season (2002) and for 63 individuals (58 females and 5 males) during the second dry season (2003). Repeatability of individual VBC scoring in a given dry season was relatively high: 79% of impalas (n = 126) that had been independently scored during consecutive days at the water points in the same year were consistently assigned to the same VBC class, and the Kappa statistics indicate a substantial agreement beyond chance between repeated scoring (Kappa test = 0.60, p < 0.001). The remaining individuals that had been assigned to different VBC classes in a given dry season were classified to their most frequent VBC class or (in case of paired number of VBC scores) were arbitrarily assigned to the class attributed at the first occasion. Removing these individuals (i.e., with a non steady VBC scores) from the dataset did not change the relationship between individual variation in body condition and explanatory variables tested in the following analyses, so we only reported results from the full dataset.

**Figure 6:**
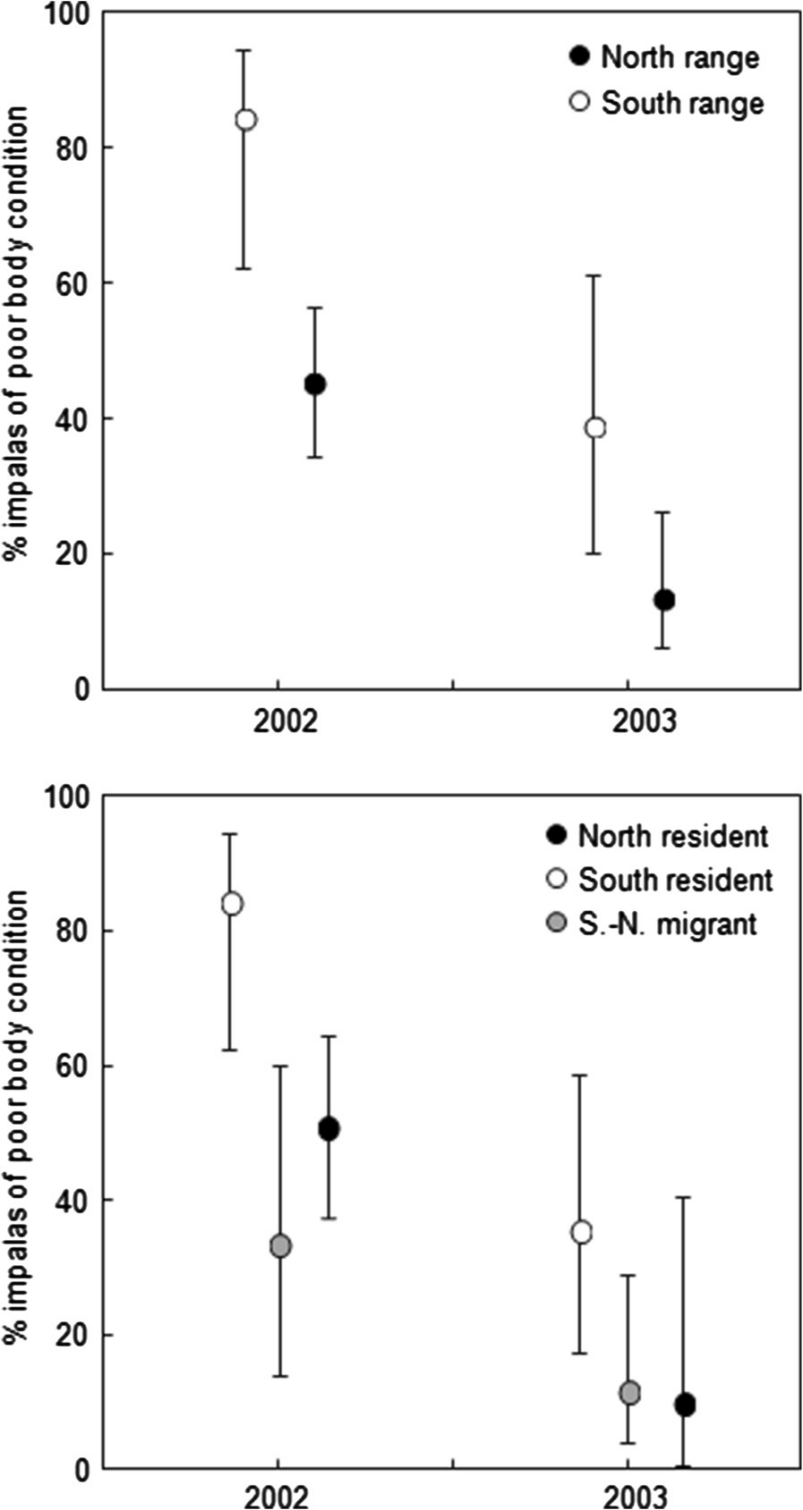
**Individual variations in body condition of impalas in relation to their dry season range and their migration strategy.** The mean proportion of impalas scored as ‘poor’ is presented ± 95% CI for the year of higher (2002) and lower (2003) population density. The best supported GLMM model of the variation of body condition among impalas present in the north or the south range during the dry season (AIC = 184.01; variance estimation of the individual random effect = 0.74; Pearson *χ*^2^ goodness-of-fit test = 110.1, ddl = 151, *P* = 0.99) revealed a significant main effects of range and year. The best supported GLMM model among impalas that used distinct migration strategies (partial data set, AIC = 157.1; variance estimation of the individual random effect = 0.76; Pearson *χ*^2^ goodness-of-fit test = 95.11, ddl = 128, *P* = 0.98) revealed a significant main effects of migration strategy and year (see Results).

Body condition of individuals was scored independently of their migration strategy classification that was not known at the time of observation. Only a portion of the impalas with a body condition score could be assigned to a seasonal range (2002: n = 92; 2003: n = 63) or a migration pattern (2002: n = 80; 2003: n = 53). In our first analysis we found a significant difference in body condition between years (LRT: *χ*^2^ = 22.78, p < 0.001) and between the dry season ranges (LRT: *χ*^2^ = 14.81, p < 0.001) (Figure 6). A significantly higher proportion of impalas were scored as poor in the first year than in the second year (difference of 2.08 ± 0.47 on a logit scale, p < 0.001). Among the individuals for which a body condition score could be assigned in both years (n = 47), a large proportion of individuals (47%), experienced an increase in body condition from low to fair between the two years (McNemar’s test for paired samples: *χ*^2^ = 20.05, p < 0.001); others had an unchanged body condition score but no individual was found to have changed from fair to low body condition between the two years. Body condition was higher among impalas present in the north range during the dry season than among those present in the south range (difference of 1.92 ± 0.52 on a logit scale, p < 0.001; Figure 6) in accordance with the between-range difference in diet quality. The between-range difference was consistent in both years with no statistically significant interaction among year and range (LRT: *χ*^2^ = 0.36, p = 0.55). We also did not detect a statistical difference in body condition between males and females (LRT: *χ*^2^ = 0.28, p = 0.59).

In our second analysis we found a significant relationship between migration strategy and body condition (LRT: *χ*^2^ = 13.47, p = 0.001) in addition to the between-year difference in body condition (LRT: *χ*^2^ = 18.29, p < 0.001) (Figure 6). Impalas that migrated from the south to the north range at the beginning of the dry season (i.e. S.-N. migrants) had a higher body condition than those that remained in the south range (i.e. south residents) (difference of 2.17 ± 0.68 on a logit scale, p < 0.001). No significant difference was found in body condition between the south to north migrants and the north residents (difference of 0.44 ± 0.62 on a logit scale, p = 0.48). The same pattern was observed in both years with no significant interaction between year and migration strategy (LRT: *χ*^2^ = 0.79, p = 0.67). The single individual that migrated from the north to the south range at the beginning of the dry season (i.e. N.-S. migrant) for which a body condition score had been attributed was classified as poor.

## Discussion

Our results provide rare evidence for a significant relationship between seasonal migration strategy and body condition in wild ungulates in relation to a difference in the quality of resources acquired between distinct seasonal ranges. The diet quality and body condition of impalas present in the north range was consistently higher than in impalas present in the south range. Individuals were consistently observed in one of the two ranges within a season but about one third of the individuals migrated from the lower (i.e. south) to the higher (i.e. north) diet quality range at the beginning of the dry season. This partial migration pattern was consistent between two consecutive years, and most individuals showed constancy to their moving strategy (migrant or resident). In both years migrants had a significantly higher body condition at the end of the dry season than individuals that remained year-round in the lower diet quality range (i.e. south resident).

The diet quality and the body condition of impalas were higher in the year of lower density suggesting a concomitant density dependent response in the nutritional and body condition of impalas. However, we did not detect any evidence for an influence of density on migration propensity and on the benefit associated with migration. First, we found no between-year difference in the proportion of migrants in the population and we observed a relatively stable migration strategy at the individual scale. Second, we did not detect a more contrasted spatial variation in diet quality or body condition between impalas that used distinct seasonal ranges or migration strategies in the year of higher density. This suggests that there is no density-dependence in the differences of body condition among individuals using these different space use tactics.

We acknowledge that the monitoring of individual space use through direct-visual identification of marked animals instead of telemetry (using radio-tracking or GPS technology) greatly limited the number of locations that we could collect and that this may be a limitation in our study. Though we cannot completely rule out the possibility for animals to have used a range elsewhere, the limited number of locations per individual was more likely due to the difficulty of identifying marked animals rather than a failure to detect marked animals during the monitoring. The entire study area was prospected to locate marked impalas but all marked impalas observed during the prospection could not be identified owing to long fleeing distance and the density of the vegetation cover. The impala population was hunted in this game ranch (by car at night throughout the study area) therefore routinely flee when approached by the observer during our monitoring.

Our data suggests that individuals were faithful between years to a seasonal range and to a migration strategy. Cautious interpretation, however, is needed because of the small number of locations collected for each individuals and the underlying assumption that they are faithful to a range during a given season. The consistency of relocation within the same seasonal range was high (96%) among individuals the most frequently observed (n = 5 to 10 locations per season), including both residents and migrants. Moreover, the attribution of a spatial tactic (from locations collected during the prospection over the study area) and a body condition score (from observation during the water point monitoring) was conducted independently through two separate protocols. Though errors in the attribution to a seasonal range or a migration strategy cannot be completely ruled out, this bias should have affected individuals independently to their body condition score, and should not have affected (apart from the introduced noise) the relationship between individual spatial tactic and body condition.

### Spatio-temporal variations in diet quality and body condition

The subdivision of the study population in two spatial units that we found is consistent with the discontinuous [28] or the clumped distribution [27] of other impala populations previously described in patchy habitats. Our study area lies on sedimentary sandstone foundations that have led to the formation of soils of varied quality [41] and distinct vegetation covers ([34], Figure 2). These two ranges are separated by a river bed (Figure 2) that though is dry most of the year may act as a natural frontier between impalas’ ranges.

Both faecal indicators that we monitored indicated a typical decrease in the diet quality of impalas during the dry season [42] as the digestibility and the nutritive value of forage decreased with the senescence and the progressive depletion of forage resources. The significantly lower diet quality that we found during the year of higher population density, together with the concurrent between-year difference in body condition and recruitment previously reported [33], provides evidence for a resource-limitation control of the performance of our study population. Forage limitation has been similarly proposed as the main regulating factor of most of the African ungulate populations that have been studied so far (white-eared kob *Kobus kob*[43]; greater kudu *Tragelaphus strepsiceros*[44]; wildebeest *Connochaetes taurinus*[45]).

A growing number of empirical studies have evidenced a relationship between the spatial heterogeneity in resource quality and the variation in performance among individuals or sub-population units (see [46] for a thorough review). In our study we found a positive co-variation in the diet quality and the body condition between the north and the south ranges, suggesting a spatial variation in habitat quality. The higher diet quality and body condition measured in impala present in the north range, in both years and in all seasons, is consistent with the spatial variation in soil quality and associated vegetation. The north range lies on alluvial terraces that form rich and well structured medium to deep profile lime soils [41]; it is mostly covered by mopane woodland, a tall, low-density but high-cover woodland with a species-rich herbaceous stratum dominated by annual grasses [34]. The south range consists of brown semi-alluvial sandy soils of lower quality; it is mostly covered by a mixed woodland with a high under-storey cover and lower species richness dominated by perennial grasses.

### Partial migration in impalas

Partial migration has been commonly reported in temperate or boreal ungulate populations (e.g. [13,15-17,20,21,23]) but it has only recently been described in a few tropical ungulates (African buffalo *Syncerus caffer*[19]; wildebeest [47]). Our findings constitute the first description to our knowledge of a partial migration pattern in an impala population.

Impala is not generally considered as a migratory species [48] but some seasonal movements of impalas between neighbouring habitats have been described. The patterns of movements were either a small seasonal shift (0.5 km) in the home range centre (Sengwa Wildlife Research Area, Zimbabwe, [28]), a periodic abandonment of the usual home range (with movements of up to 10 km) during periods of severe food shortage (Serengeti NP, Tanzania, [49]), or more extensive movements (5-50 km) leading to distant wet and dry season ranges (Southern low-veld and Mana Pools NP, Zimbabwe [50,51]). In our study about one third of the impala population shifted between two adjacent ranges and this pattern was consistent during two consecutive years. Migration in ungulates is usually viewed as long-distance movements. However, following Ball et al. (2001) [13], we considered that short-distance seasonal movements are properly termed ‘migration’ if there is a clear shift between spatially disjunct seasonal ranges, regardless of how close these might be.

Such a partial migration has important consequences for the degree of mixing within a population that has not been reported for impala so far. Though instability in group membership has long been recognised in impala [50], the social structure has been reported to be highly cohesive year-round at the clan level [30]. A high level of association has been found between members of the same clan with only occasional transfer (2% per annum [28]) of females between adjacent clans. Conversely in our study about one third of individuals (both females and males) changed their seasonal range. Interestingly we observed no segregation between resident and migrant impalas, which were found in mixed herds while sharing the same seasonal range similar to that described in another gregarious ungulate species, the elk *Cervus elaphus*[22]. Social constraints to individual exchanges between population spatial units have been reported in various gregarious ungulate species [1,15]. In impala, the female social status as well as the mother-offspring bond after weaning is of little significance and agonistic interactions between females are scarce [30]. In Southern Africa, most males are only territorial for a few weeks during the time of the annual rut (i.e. in April) and generally associate with females in mixed herd throughout most of the year [48]. The seasonal social mixing that we observed may have been facilitated by the typical unstable group units of impalas with a fluid membership and by the absence of dominance hierarchy [30]. The period of rut did not coincide with the timing of migration (migrants were observed for the first time in their dry season range in July). This suggests that migration was not associated with a mating dispersal in this population.

Several hypotheses have been proposed to explain partial migration in animals ([12,15,52]). In our study the direction of migration at the onset of the dry season was correlated with the difference in diet quality between the two ranges for almost all migrants. Access to higher quality forage is generally considered to be one of the major driving forces of seasonal migration [9-12]. At the beginning of the wet season most migrants however returned to their previous wet season range in the south associated with a relatively lower diet quality. The differences in diet quality between seasons were more contrasted than the differences between ranges. In tropical ecosystems food resource acquisition is likely to be much less constraining during the wet season when forage is of higher quality and availability. The nutritional cost associated with the relatively lower forage quality of the south range should be lower in the wet season and may be balanced by other benefits. The high fidelity to a summer or winter range documented in temperate ungulates (e.g. [18]) or to a wet season range in a tropical ungulate (e.g. [47]) is considered to provide benefits related to familiarity with the location of resources, predators or conspecifics [53]. The impalas that we monitored had all been introduced at the same release site located in the south range (Figure 1) which therefore may be more familiar than the north range and may provide some predator-avoidance benefits. This is supported by the fact that migrants were first observed in the south range in November just before the calving period (occurring in December), a time when females may be particularly sensitive to the risk of predation [9]. It also has to be noted that the migration movements performed by these impalas are relatively small (a few kilometres). This decreases the risks and associated costs of migration movements (increased energy expenditure or predation exposure along migration pathways) and probably contributes to the benefit of migration observed in this population.

Translocation has been shown to alter the movement patterns of wild ungulates only during the first years after introduction into a novel environment [54]. In our study, monitoring started more than two years after the last impalas had been introduced in the study area. The fact that most individuals used the same seasonal range and displayed the same migration strategy for two consecutive years suggests that the translocated impalas had adopted a stable space-use pattern and that the shift in seasonal range that we observed were actual migrations rather than dispersive or exploratory movements.

Access to surface water has been proposed as a potential determinant of migration in tropical ungulates [19]. Impala is generally considered to be highly water-dependent [48] and to be forced to make daily excursions for water during the dry season [28]. In our study, despite the drastic reduction in the distribution of water availability between the wet and the dry seasons (see the Methods), we observed no marked changed between the wet and the dry seasons in the distribution of impalas over the north or the south ranges (Figure 3). Access to drinking water did not appear to have shaped migratory behaviour since permanent water sources were equally available in both the north and the south ranges, and impalas were always located at <3 km of a water source. Moreover our continuous monitoring of marked impalas at water points during consecutive days indicates that, at the end of the dry season, impalas were visiting water points only every two or three days rather than daily [33].

## Conclusions

In our study, migration was associated with a benefit in terms of access to a range providing, on average, a higher diet quality and higher performance in terms of body condition. Interestingly, the increase in forage competition in the north range associated with the influx of migrants during the dry season did not offset the advantages of access to forage of higher quality as revealed by the higher diet quality measured in the north range. Migrants that had spent the wet season in the lower quality south range had a similar body condition at the end of the dry season to the individuals that remained year-round in the higher-quality range (i.e. north resident). This suggests that migrants did not experience a seasonal carry-over effect, i.e. an influence of the habitat quality in the preceding season [55]. This result is consistent with the low potential of impalas to store fat reserves [56]. Alternatively, there might have been some individual differences in resource acquisition during the wet season among impalas present in the south range, and only individuals of higher phenotypic quality may have decided to migrate. Investigating the relationship between the individual physiologic, genetic or social characteristics and their migration propensity would be a relevant direction for future researches.

Few studies have explored so far the relationship between migration strategies and fitness-related traits in ungulates [12,55]. In two recent studies, migratory females had either a higher calving success than residents (in reindeers *Rangifer tarandus*[17]) or a higher fecundity and calf weight (in elk *Cervus elaphus*[18]). In contrast, a higher mortality rate (in elk [18]; and in mule deer *Odocoileus hemionus*[16]) or lower pregnancy and recruitment rates (in elk [23]) were found in migratory compared to resident females. High mortality risk during the migratory route, associated with energy travelling expenditure, injuries or higher predation exposure, have been documented in a few other cases (in elk [21]; in reindeers [17]). Such a distinct trade-off between survival and reproduction between co-existing migrants and residents, if it is confirmed for other ungulates, would be crucial for shaping the population dynamics of partially migratory ungulate populations [52] and for predicting their sensitivity to migration disruption [23,55]. The individual variation in space use tactics increases the plasticity of ungulate population to changes in environmental conditions. An important aspect of future research, with high relevance to conservation of migratory ungulates, would be to examine the individual flexibility to switch between migration tactics in response to change in habitat quality, predation or human disturbance.

## Authors’ contributions

NG designed the study and collected all the data. NG analysed the data and performed the statistical analysis; PC designed and performed the diet quality analysis. NG wrote the manuscript; PC provided editorial advice. Both authors read and approved the final manuscript.
